# Tailoring TiO_2_ Nanotube Arrays via Anodization Voltage and Thermal Processing: A Parametric Design Roadmap for Customizable Biomedical Applications

**DOI:** 10.3390/pharmaceutics18101241

**Published:** 2026-09-30

**Authors:** Ildiko Peter, Alex-Barna Kacsó, László Jakab-Farkas

**Affiliations:** 1 Department of Industrial Engineering and Management, George Emil Palade University of Medicine, Pharmacy, Science, and Technology of Targu Mures, Gheorghe Marinescu 38, 540142 Târgu Mureș, Romania; ildiko.peter@umfst.ro; 2 Doctoral School of I.O.S.U.D., George Emil Palade University of Medicine, Pharmacy, Science, and Technology of Targu Mures, Gheorghe Marinescu 38, 540142 Târgu Mureș, Romania; 3 Faculty of Technical and Human Sciences, Sapientia Hungarian University of Transylvania, 540485 Târgu Mureș, Romania; jflaci@ms.sapientia.ro

**Keywords:** TiO_2_ nanotubes, electrochemical anodization, drug carrier reservoir, morphological control, thermal annealing, capillary stress, resistance, biomedical implants

## Abstract

**Background/Objectives:** Titanium and its flagship alloy (Ti6Al4V) represent the clinical gold standard for orthopedic and dental implants; however, optimizing their surface architecture to serve as high-capacity, reliable platforms for localized therapeutic delivery remains a significant clinical challenge. In this study, we aim to establish a systematic design roadmap linking surface engineering parameters directly to functional drug delivery performance. **Methods:** To achieve this, a comparative investigation was performed to map the electrochemical growth kinetics, voltage-driven morphological evolution, and processing limits of TiO_2_ nanotube (TNT) arrays developed on commercially pure titanium (CP-Ti) and Ti6Al4V substrates in an ethylene glycol-based electrolyte containing NH_4_F. Anodization was carried out at three different potentials (40 V, 50 V, and 60 V) for 30 min, followed by structural and statistical evaluation. **Results:** Scanning electron microscopy (SEM) combined with Gaussian size-distribution modeling confirmed a strong linear correlation between applied potential and pore dimensions, expanding mean diameters from 59 nm to 88 nm on CP-Ti and from 56 nm to 96 nm on Ti6Al4V. Current–time (I-t) transient analysis revealed distinct growth kinetics, with Ti6Al4V exhibiting enhanced current fluctuations driven by the differential oxidation rates of its dual-phase (α + β) microstructure. Furthermore, post-anodization thermal annealing at 450 °C is identified as a necessary structural processing step to stabilize the amorphous layers into crystalline polymorphs, providing the essential capillary stress resistance required to prevent film delamination during fluidic drug loading (MgSO_4_ simulation). **Conclusions:** By establishing a comprehensive parameter-property matrix, this study provides a practical design roadmap that categorizes specific anodization regimes for tailored pharmaceutical and biomedical functions, ranging from direct osteoblast adhesion (40–50 V) to superhydrophilic architectures structurally optimized for high-capacity drug delivery (60 V).

## 1. Introduction

The clinical performance of orthopedic and dental endoprostheses heavily relies on the interfacial events occurring between the synthetic implant surface and the surrounding biological tissue. Although metallic titanium and its multi-phase product, Ti6Al4V, dominate the biomedical implant market due to their superior strength-to-weight ratio and corrosion resistance [1], their native, bioinert oxide film often fails to induce rapid peri-implant bone formation. Unmodified titanium surfaces typically show limited bioactive sensitivity, which can prolong patient recovery times and increase the risk of aseptic implant failure [2]. Consequently, engineering nanostructured surface features has emerged as a key pathway to accelerate cellular signaling, protein adsorption, and overall osseointegration [3].

To bridge this bio-functional gap, electrochemical anodization has gained widespread attention as a versatile and scalable surface modification technique. This method enables the spontaneous formation of self-organized titanium dioxide (TiO_2_) nanotube arrays (TNTs) that closely mimic the natural nanoscale topography of the human bone matrix [4]. In addition to topographic cues, the post-synthesis phase transformation of these amorphous oxide layers via thermal annealing—converting them into crystalline anatase or rutile polymorphs—is widely recognized for optimizing surface energy and superhydrophilic characteristics, both of which are essential for early-stage osteoblast attachment and for facilitating the instantaneous capillary infiltration of aqueous therapeutic formulations [5].

Despite extensive documentation on TNT synthesis, translating these nanostructures into clinical practice faces a significant design challenge: different medical applications demand fundamentally different structural topographies. For instance, direct osteoblast proliferation requires narrow, highly uniform pore geometry, whereas high-capacity fluid hosting or local antimicrobial agent delivery calls for significantly larger internal volumes to achieve clinically effective drug loading and sustained release kinetics [6]. The physical dimensions of these nanostructures are largely determined by the applied potential and the underlying substrate composition. Comparing growth dynamics on commercially pure titanium (CP-Ti) against the dual-phase (α + β) Ti6Al4V alloy is particularly important, as the selective dissolution of alloying elements (Al and V) drastically alters local current distribution and array uniformity.

Furthermore, hollow nanotubular channels serve as idealized micro-reservoirs, ensuring structural integrity and that proper phase transformation via thermal processing remains an essential requirement before any liquid-phase functionalization or clinical deployment. Specifically, the amorphous native tubes are highly susceptible to severe capillary drying stresses during drug encapsulation, which often leads to catastrophic film delamination [7]. Thus, establishing robust thermo-mechanical stability is an absolute prerequisite for utilizing these matrices as reliable drug carriers. While the fundamental synthesis of TiO_2_ nanotube arrays via electrochemical anodization is well-documented in the literature [8,9], the systematic optimization of these structures specifically as high-capacity pharmaceutical frameworks remains underexplored. The explicit novelty of this study lies in the comprehensive comparative evaluation of CP-Ti and the heterogeneous Ti6Al4V alloy under combined high-voltage conditions (up to 60 V) and thermal annealing. By formulating a predictive parameter-property matrix, this work directly correlates synthesis variables with the essential physical prerequisites for localized drug delivery—such as structural void volume and superhydrophilic capillary infiltration capacity—thereby bridging the gap between basic material synthesis and advanced physical pharmaceutics.

To address these fundamental questions, this study explores the voltage-governed growth behavior and structural characteristics of TiO_2_ nanotube arrays synthesized on both CP-Ti and Ti6Al4V substrates across a potential range of 40–60 V. Rather than focusing on a single, isolated application, the main objective is to clarify how electrical parameters and substrate microstructures interact to affect pore dimensions, growth kinetics, and layer uniformity. By establishing these fundamental process–structure relationships, this research seeks to construct a predictive framework that guides the selection of specific anodization regimes for targeted biomedical functions while clarifying the structural role of thermal processing in overcoming capillary-induced failure, thereby establishing a mechanically robust physical foundation for localized drug delivery systems.

## 2. Materials and Methods

This section outlines the experimental framework employed to build, structurally optimize, and evaluate the voltage-dependent behavior of bioactive TiO_2_ nanotube arrays. The systematic workflow spans initial substrate preparation and electrochemical anodization across multiple potentials (40–60 V), post-synthesis thermal processing for phase transformation, morphological characterization via SEM and statistical Gaussian modeling, and subsequent structural integrity assessment under fluid exposure conditions. These fluid exposure protocols were specifically designed to simulate the physical stresses of therapeutic loading and solvent evaporation.

### 2.1. Substrate Preparation and Pre-Treatment

CP-Ti and Ti6Al4V samples were prepared as disc-shaped samples with a uniform diameter of 16 mm and a thickness of 3 mm. To ensure a standardized starting topography for the anodization process, all samples were subjected to a rigorous manual grinding and polishing protocol. Initially, the surfaces were mechanically ground using sequential grades of silicon carbide (SiC) abrasive papers (320, 600, and 1200 grit; QATM, Kuchl, Austria). Subsequently, the samples were manually polished on specialized polishing cloths (Touch Lam, Lam Plan, Gaillard, France) using VOC-free diamond pastes with progressively decreasing particle sizes (6 µm, 3 µm, and 1 µm; Neodia Biodiamant, Lam Plan, Gaillard, France) until a smooth, mirror-like finish was achieved. To strictly prevent abrasive cross-contamination, the samples were thoroughly rinsed between each consecutive grinding and polishing step with distilled water, which was produced in-house using a water distillation system (Typ 2001/4; GFL, Burgwedel, Germany) between each consecutive grinding and polishing step. Finally, all polished samples were washed in absolute ethyl alcohol (Chemical Company, Iași, Romania) to remove any residual polishing paste or organic contaminants, and subsequently dried in ambient air before the electrochemical anodization.

### 2.2. Electrochemical Anodization

The development of TiO_2_ nanotube arrays on the prepared CP-Ti and Ti6Al4V substrates was carried out using a conventional two-electrode electrochemical cell setup. Importantly, freshly polished, individual titanium samples were used for each specific parameter set to ensure independent measurements; no sample was subjected to multiple anodization cycles. The titanium sample served as the working electrode (anode), while a copper (Cu) electrode (dimensions: 20 mm in diameter and 3 mm in thickness) was utilized as the counter electrode (cathode). During the process, the immersion depth into the electrolyte was strictly controlled at 10 mm for the titanium anode and 12 mm for the copper cathode. The distance between the anode and the cathode was maintained at a constant 20 mm for all experiments.

Anodization was performed in an organic electrolyte composed of ethylene glycol (Honeywell Riedel-de Haën, Seelze, Germany) containing 0.5 wt% ammonium fluoride (NH_4_F, Honeywell Fluka, Seelze, Germany) and 2 wt% distilled water [8]. The process was conducted at ambient temperature without any external cooling or heating systems. This ambient-temperature electrochemical synthesis represents a highly scalable and cost-effective manufacturing approach for producing pharmaceutical implant coatings. To investigate the effect of voltage on the nanotubular morphology, the anodization was carried out at three distinct constant potentials: 40 V, 50 V, and 60 V. A programmable DC power supply (9184B, B&K Precision, Yorba Linda, CA, USA) was used to drive the reaction. To prevent sudden current surges and ensure uniform pore nucleation, the voltage was initially ramped up from 0 V to the target potential at a constant sweep rate of 1 V/s.

Once the target voltage was reached, the anodization process was held constant for exactly 30 min for each sample. Immediately after the completion of the electrochemical process, the anodized samples were promptly removed from the electrolyte and thoroughly washed with absolute ethyl alcohol to halt the reaction and remove any residual viscous electrolyte or fluoride ions. Finally, the samples were covered to protect them from dust and allowed to dry completely at room temperature.

### 2.3. Post-Anodization Thermal Processing

To induce the crystalline phase transition of the TiO_2_ nanotubes from an amorphous state to the bioactive anatase phase, the as-anodized samples were subjected to a thermal annealing process in a muffle furnace (N 11/HR, Nabertherm GmbH, Lilienthal, Germany). The samples were placed into the furnace at ambient temperature and heated to a target temperature of 450 °C using a controlled heating ramp to prevent thermal stress and delamination of the nanotubular layer [5]. This controlled crystallization step is hypothesized to be the fundamental determinant for establishing the mechanical robustness required for safe drug encapsulation. Once the target temperature was reached, the isothermal plateau was maintained for a defined duration of 2 h. Following the annealing period, the furnace was switched off, and the samples were allowed to cool slowly to room temperature inside the sealed chamber for approximately 19 h before removal.

### 2.4. Surface Characterization and Image Statistics

The surface morphology and topographical features of the TiO_2_ nanotubes were analyzed using a SEM (Zeiss Evo40, Carl Zeiss, Oberkochen, Germany) operating at an accelerating voltage of 25 kV. The structural dimensions, specifically the inner pore diameters of the nanotubes, were quantitatively analyzed from the obtained SEM micrographs using the open-source ImageJ software (version 1.53, National Institutes of Health, USA). To evaluate the pore size distributions and voltage-dependent dimensional boundaries, statistical analysis was performed by fitting Gaussian distributions to the measured diameter data sets.

The surface roughness of the samples was evaluated using a mechanical stylus profilometer (MITUTOYO SJ-310, Mitutoyo Corporation, Kawasaki, Japan). To accurately assess the topographical impact of the electrochemical treatment and the subsequent thermal annealing, the average surface roughness parameter R_a_ was measured in triplicate (n = 3) on both the untreated (bare metal) regions, as well as on the anodized nanotubular regions, both before and after the thermal treatment.

Surface wettability, a pivotal factor for initial biological response, was determined at room temperature using the static sessile drop technique. To evaluate the exact impact of the crystalline transition, these measurements were systematically performed on the nanotubular surfaces both before and after the post-anodization thermal annealing process. A constant droplet volume of 4 µL of distilled water was carefully dispensed onto the sample surfaces utilizing a MicroPette Plus Autoclavable Pipettor (DLAB Scientific, Beijing, China). To ensure dynamic consistency across all measurements, macro photographs of the droplets were captured exactly 3 s after initial contact with the surface. The resulting contact angles were then precisely extracted and calculated from digital images using the angle measurement tool within the ImageJ software. These wettability metrics provide direct insight into the fluid-matrix interfacial energetics that govern liquid drug infiltration and subsequent release behaviors.

To determine the statistical significance of the differences observed in pore diameters, surface roughness (R_a_), and contact angles across the varying anodization potentials, data were analyzed using a one-way analysis of variance (ANOVA) followed by Tukey’s post hoc test. A *p*-value of <0.05 was considered statistically significant.

### 2.5. Fluid Exposure and Structural Stability Assessment

To evaluate the mechanical robustness and structural tolerance of the nanostructure against capillary stress during liquid functionalization, comparative fluid exposure tests were realized on both as-anodized (amorphous) and thermally annealed (crystalline) TiO_2_ nanotube arrays. A 0.1 M MgSO_4_ model solution was prepared by dissolving magnesium sulfate heptahydrate crystals (MgSO_4_·7H_2_O; Chemical Company, Romania) in distilled water via manual agitation at room temperature until complete dissolution was achieved [10]. This specific salt solution was selected as a safe, inorganic proxy to effectively mimic the osmolarity, fluid dynamics, and crystallization stresses typically exerted by liquid pharmaceutical formulations within nanoporous reservoirs. Controlled droplets (10 µL) of the solution were carefully applied onto the centers of the nanotubular surfaces using a micropipette.

To investigate the thermal evaporation dynamics and drying-induced stress, two distinct thermal drying protocols were evaluated at 60 °C:

- **Convective Oven Drying:** Samples were placed inside a preheated laboratory drying oven to achieve uniform ambient heating surrounding the entire sample.
- **Bottom-Heated Plate Drying:** Samples were positioned on a heated metallic block to supply direct conductive thermal energy exclusively from the substrate base, preventing rapid top-surface evaporation.

Following complete liquid evaporation under both thermal conditions, the structural integrity of the nanotube arrays was systematically evaluated. The amorphous (as-anodized) layers consistently exhibited severe failure, leading to complete delamination from the metallic substrate regardless of the drying method. In contrast, the thermally annealed (crystalline) samples maintained structural adhesion to the substrate, exhibiting only localized microscopic surface cracks. Surface morphology and film adhesion were observed using optical and scanning electron microscopy (Zeiss Evo40, Carl Zeiss, Oberkochen, Germany).

## 3. Results and Discussion

This section systematically presents and evaluates the morphological, topographical, and structural properties of the developed TiO_2_ nanotube arrays engineered as architectural frameworks for localized drug delivery. The morphological evolution, layer uniformity, and pore size distributions are first established using SEM analysis and Gaussian statistical modeling. Subsequently, the influence of anodization voltage and thermal annealing on surface roughness (R_a_) and wettability (contact angle) is analyzed. Finally, the mechanical robustness and structural integrity of the nanotubular layers are evaluated under fluid exposure and thermally induced capillary stress conditions, simulating the physical demands of a therapeutic agent loading phase.

### 3.1. Nanotube Morphology and Voltage-Driven Geometric Evolution

The electrochemical growth mechanism and the subsequent morphological evolution of the anodized layers were systematically investigated as a function of the applied anodization potential (40 V, 50 V, and 60 V) and substrate composition (CP-Ti vs. Ti6Al4V alloy) to determine the optimal matrix geometry for drug encapsulation.

#### 3.1.1. Electrochemical Growth Dynamics

To understand the nucleation, self-organization, and growth kinetics of the TiO_2_ nanostructures, transient current-intensity responses during the 30 min anodization process were recorded for both substrates.

**Figure 1:**
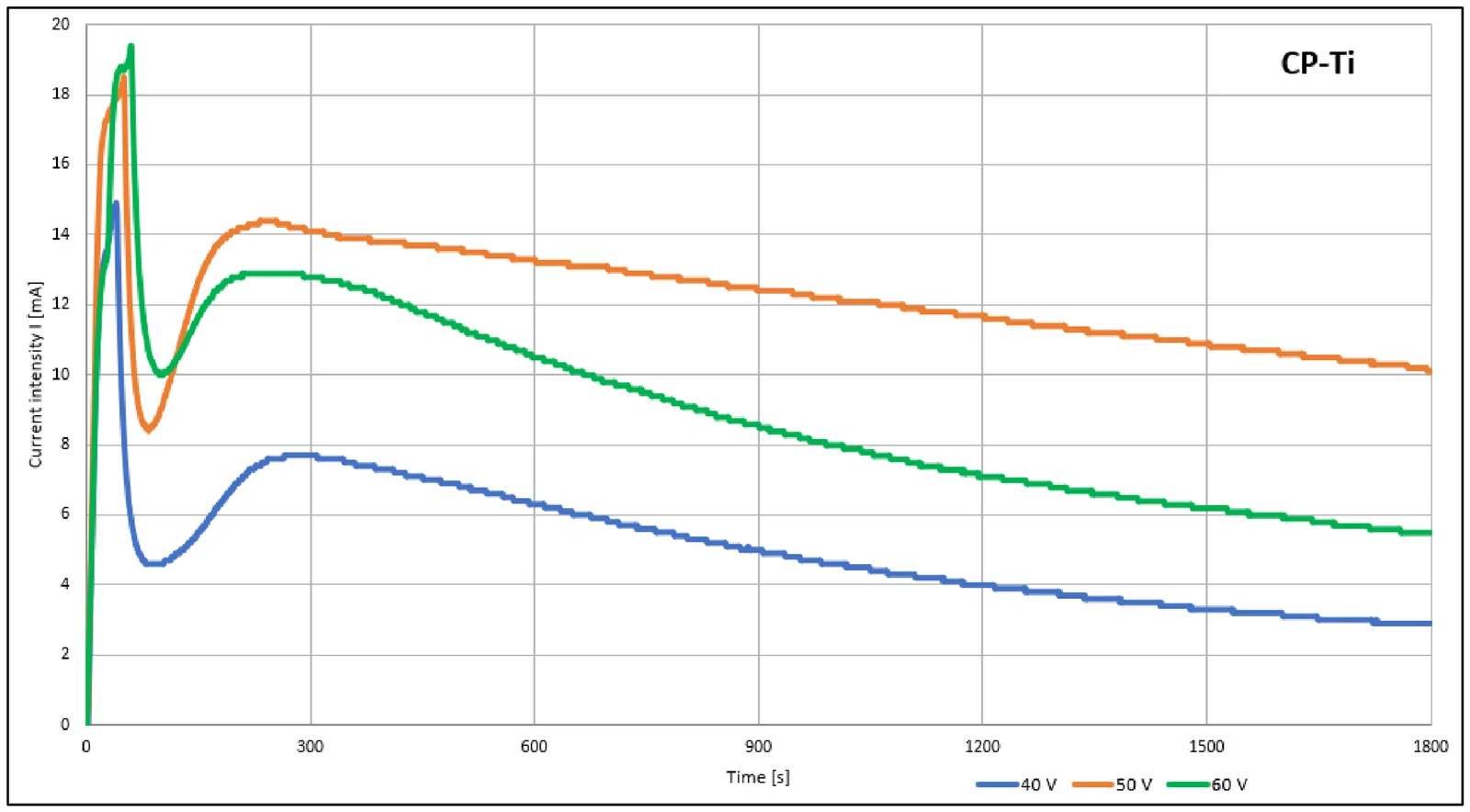
Current intensity transients during anodization of CP-Ti at 40, 50, and 60 V.

**Figure 2:**
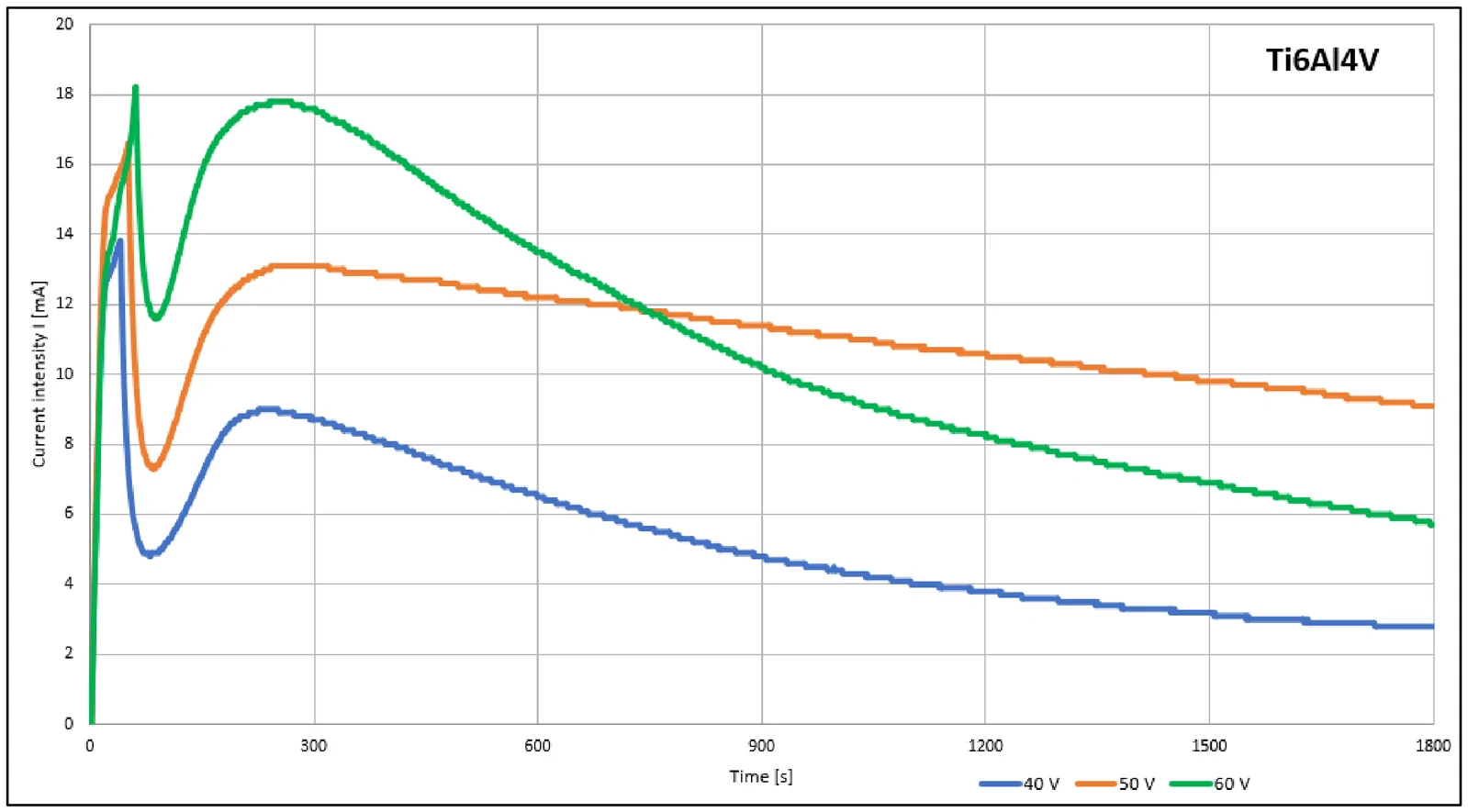
Current intensity transients during anodization of Ti6Al4V at 40, 50, and 60 V.

As demonstrated in Figure 1 and Figure 2, the current intensity–time (I-t) transients for both CP-Ti and Ti6Al4V alloy exhibit the classic three-stage behavior characteristic of self-organizing anodization in fluoride-containing organic electrolytes [4,7]:

- **Stage I (Initial Passivation & Ramping):** In the first 40–60 s of the process, a sharp current peak (14–19 mA) is recorded, driven by the linear voltage ramp (1 V/s). This is immediately followed by a rapid drop in current intensity, attributed to the formation of a compact, high-resistance native oxide barrier layer (TiO_2_) on the metallic surfaces.
- **Stage II (Pore Nucleation):** Following the initial minimum point, the current experiences a temporary, characteristic surge. This rise is driven by localized chemical dissolution of the oxide layer via fluoride ions (F^−^). The field-assisted attack of F^−^ ions concentrates at surface defects, triggering random pore nucleation and localized pitting. These initial pitting sites act as the primary templates for the nanoporous network, ultimately determining the spatial distribution and density of the future drug-loading cavities.
- **Stage III (Quasi-Steady-State Growth):** Eventually, the current intensity stabilizes into a gradual decay toward a quasi-steady state. This balance signifies dynamic equilibrium between field-assisted oxide growth at the metal/oxide interface and chemical dissolution at the oxide/electrolyte interface, yielding continuous, highly organized nanotubular structures.

A distinct potential-dependent trend is observed when evaluating the transient profiles. Increasing the anodization voltage from 40 V to 50 V and 60 V significantly elevates the initial nucleation peak and accelerates dissolution kinetics due to stronger electric fields. Notably, for both substrates, the 50 V electrolyte current maintains a higher plateau throughout the steady-state phase than the 40 V system. However, at 60 V, high ionic resistance within the thickening nanotube channels and partial pore-wall dissolution result in a steep current decline after 300 s, leading to a steady-state current lower than that of the 50 V trial.

This strong dependence of the electrochemical dynamics on the applied potential directly dictates the final architecture of the nanotube arrays. The underlying physical driving force—the equilibrium between field-assisted oxidation and fluoride-induced dissolution—means that higher electrostatic fields at 60 V accelerate the downward dissolution at the pore bottoms, significantly expanding the internal pore diameter. From a pharmaceutical engineering perspective, this potential-driven morphological tuning is a highly effective tool: adjusting the voltage enables precise control of the structural encapsulation volume, facilitating a tailored shift from narrow-pore matrices theoretically suited for sustained release to wide-pore frameworks designed to maximize high-capacity local drug delivery potential [6,11].

Furthermore, clear compositional differences emerge between the two substrates. The current transients for the Ti6Al4V alloy (Figure 2) exhibit a substantially higher secondary current peak (reaching nearly 18 mA at 60 V) compared to the smoother profile of CP-Ti (Figure 1). This divergence reflects complex anodization kinetics induced by the two-phase (α + β) microstructure of the alloy, where the vanadium-rich β phase dissolves at a faster rate than the aluminum-rich α matrix, modulating local current distribution during early self-organization [4,7].

As a result of these differential dissolution rates, the dual-phase nature of Ti6Al4V inherently influences the kinetic evolution of the oxide layer compared to the single-phase CP-Ti substrate. While both materials successfully form ordered arrays, identifying these kinetic variations is essential for ensuring reproducible therapeutic loading capacities across different standard medical implant alloys.

#### 3.1.2. Topographical and Microstructural Evolution

**Figure 3:**
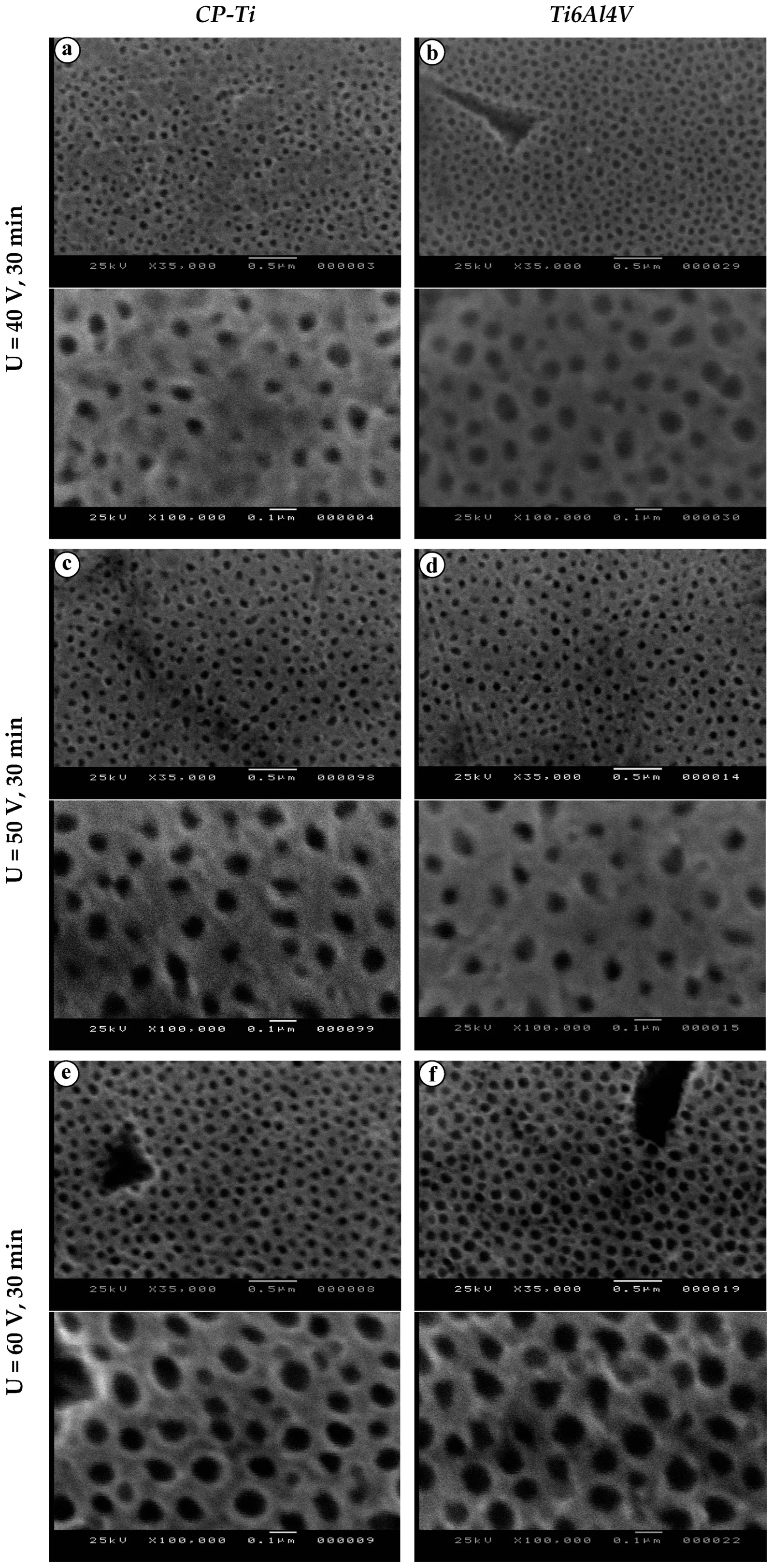
SEM surface micrographs of the anodized oxide layers obtained at different potentials for 30 min: (**a**) CP-Ti at 40 V, (**b**) Ti6Al4V at 40 V, (**c**) CP-Ti at 50 V, (**d**) Ti6Al4V at 50 V, (**e**) CP-Ti at 60 V, and (**f**) Ti6Al4V at 60 V.

The surface topography and microstructural features of the oxide layers formed after 30 min of anodization were directly evaluated using SEM. As demonstrated in Figure 3, highly ordered, self-organized TiO_2_ nanotubular architectures were successfully synthesized across all experimental conditions. The dual-magnification micrographs provide a comprehensive overview of both the macro-scale spatial homogeneity (35,000× magnification) and the localized individual pore wall geometry (100,000× magnification).

Qualitative visual analysis of the SEM micrographs (Figure 3) reveals a clear, potential-driven geometric evolution in the surface architecture. At 40 V (Figure 3a,b), the surface exhibits a densely packed array of narrow, fine pore openings with relatively thin tubular walls. Increasing the anodization voltage to 50 V (Figure 3c,d) and 60 V (Figure 3e,f) induces a progressive lateral expansion of the nanotube channels, yielding visibly wider pore diameters (*p* < 0.05), enhanced wall definition, and more pronounced inter-tubular boundaries. This lateral expansion is a direct consequence of the intensified field-assisted oxide dissolution at higher voltages. For local drug delivery systems, this geometric evolution is highly advantageous: the wider, well-defined pore openings generated at 60 V minimize steric hindrance during the infiltration of therapeutic fluids, thereby providing an optimized structural template to facilitate more efficient and homogenous drug loading across the implant surface [11].

Furthermore, a distinct structural divergence is observable when comparing the two substrate materials:

- **CP-Ti substrate (Figure 3a,c,e):** The nanotube arrays maintain an exceptionally uniform, continuous, and highly symmetrical hexagonal-like close-packed arrangement across the entire analyzed region, indicating balanced field-assisted oxidation and chemical dissolution kinetics. This high degree of symmetry provides a highly predictable and consistent void volume, which is an ideal structural trait for achieving stable, zero-order release kinetics in pharmaceutical applications.
- **Ti6Al4V alloy (Figure 3b,d,f):** The oxide film displays localized topographical disruptions, subtle pore packing disorders, and minor variations in tube circularity. High-magnification views highlight small structural boundary defects, which originate from the heterogeneous dual-phase (α + β) microstructure of the titanium alloy. During electrochemical anodization, the vanadium-rich β phase undergoes preferential, accelerated dissolution compared to the aluminum-rich α matrix, altering local current distributions and subtly distorting the ideal self-organization process.

ImageJ software. To ensure statistical reliability and high precision, a sample size of N = 50 individual pores per sample condition was measured from high-magnification SEM images (100,000×). For each analyzed pore, both the major (length) and minor (width) axes were evaluated to account for any structural ellipticity or minor distortions.

To quantitatively validate these visual observations and analyze the geometric evolution of the pore openings, precise dimensional measurements were conducted using.

**Figure 4:**
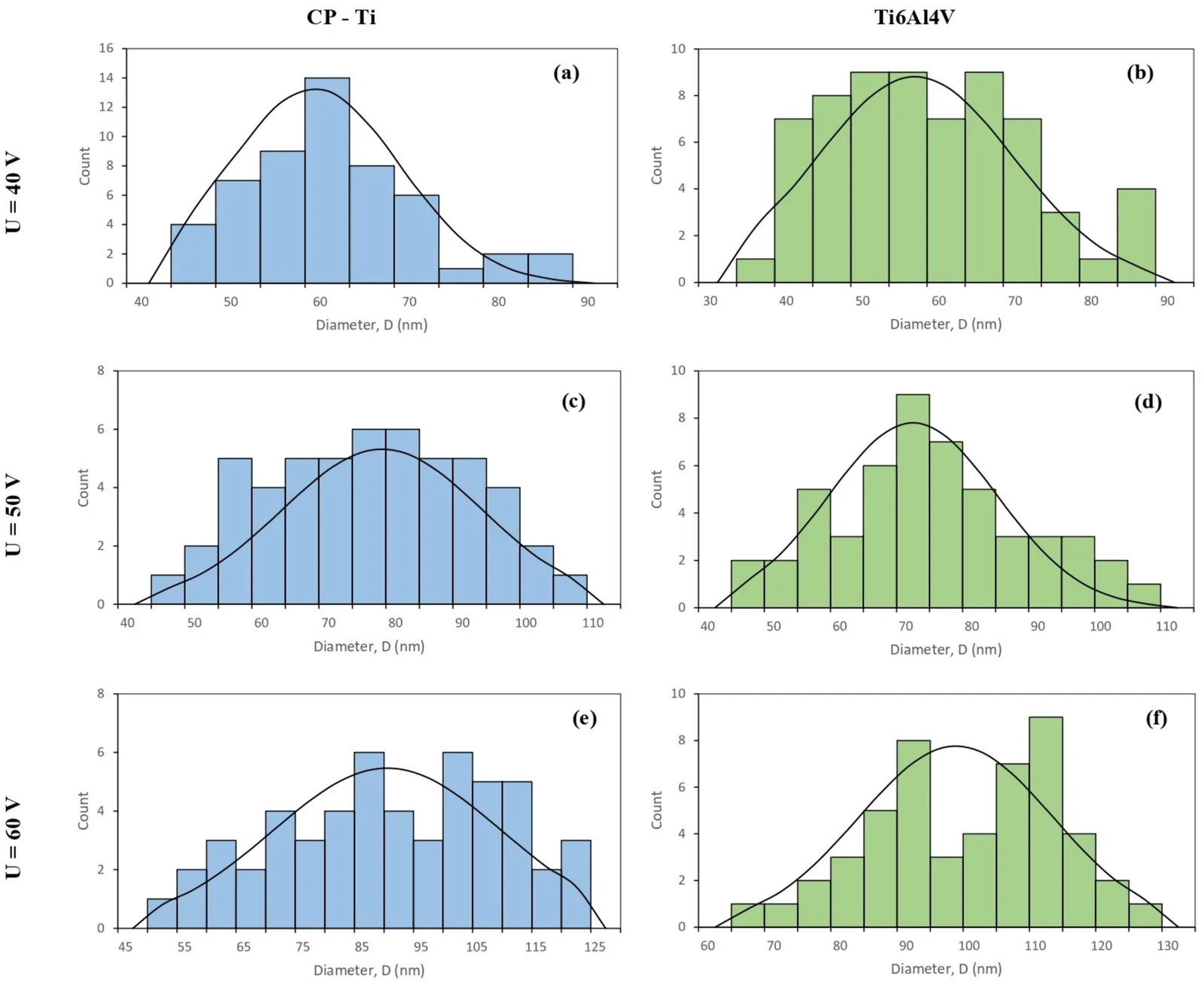
Pore diameter distribution histograms with fitted Gaussian curves for TiO_2_ nanotube arrays: (**a**) CP-Ti at 40 V, (**b**) Ti6Al4V at 40 V, (**c**) CP-Ti at 50 V, (**d**) Ti6Al4V at 50 V, (**e**) CP-Ti at 60 V, and (**f**) Ti6Al4V at 60 V.

Frequency distribution histograms constructed from these ImageJ measurements were subsequently fitted with Gaussian distribution functions to evaluate the degree of monodispersity and spatial order, as presented in Figure 4.

The fitted Gaussian profiles clearly capture the geometric shift and structural uniformity driven by both the anodization potential and the substrate matrix:

- **Voltage-Driven Translation:** As the potential increases from 40 V to 50 V and 60 V, the centroids (peaks) of the Gaussian curves systematically shift toward higher diameter values for both materials. At 40 V (Figure 4a,b), the pore population is tightly concentrated in the lower range (50–60 nm). Elevating the voltage to 50 V (Figure 4c,d) and 60 V (Figure 4e,f) broadens the base and translates the dominant peak toward ~75–80 nm and ~90–100 nm, respectively.
- **Substrate-Dependent Distribution:** The width and amplitude of the fitted Gaussian curves reflect the dimensionally monodisperse features of the nanotubes. The histograms for CP-Ti (Figure 4a,c,e) demonstrate higher, narrower bell-shaped curves with clear central modes, confirming a highly uniform pore size population. In contrast, the Gaussian distributions for the Ti6Al4V alloy (Figure 4b,d,f) show visibly broader profiles with lower peak heights, indicating a wider scatter in pore diameters caused by localized dissolution rate differences between the α and β phases.

Additionally, the close alignment between the peak center of the Gaussian fits and the calculated statistical metrics (mean, median, and mode) confirms a well-behaved, symmetric normal distribution across all experimental conditions. Importantly, repeating these measurements after thermal annealing at 450 °C confirmed that the Gaussian distribution profiles remained completely unchanged, supporting that the phase transformation to anatase does not alter the geometric pore spread. This geometric preservation post-annealing is vital, as it ensures that the carefully tailored drug-loading capacities designed during the initial anodization phase are not compromised by the thermal processing required to mechanically stabilize the matrices.

In addition to evaluating the surface pore dimensions, the cross-sectional layer thickness (nanotube length) was quantitatively investigated using SEM to determine the vertical growth kinetics of the anodic oxide films. To expose the internal nanotubular architecture, a controlled mechanical scratch was introduced on the sample surface. Cross-sectional SEM imaging was subsequently performed by tilting the stage at a 45° angle relative to the electron beam, and appropriate trigonometric geometric correction factors $\left(\frac{1}{\cos(45{}^{\circ})}\right)$ were applied to convert the apparent tilted depth measurements into true layer thickness (n = 20 individual points measured per sample condition).

**Figure 5:**
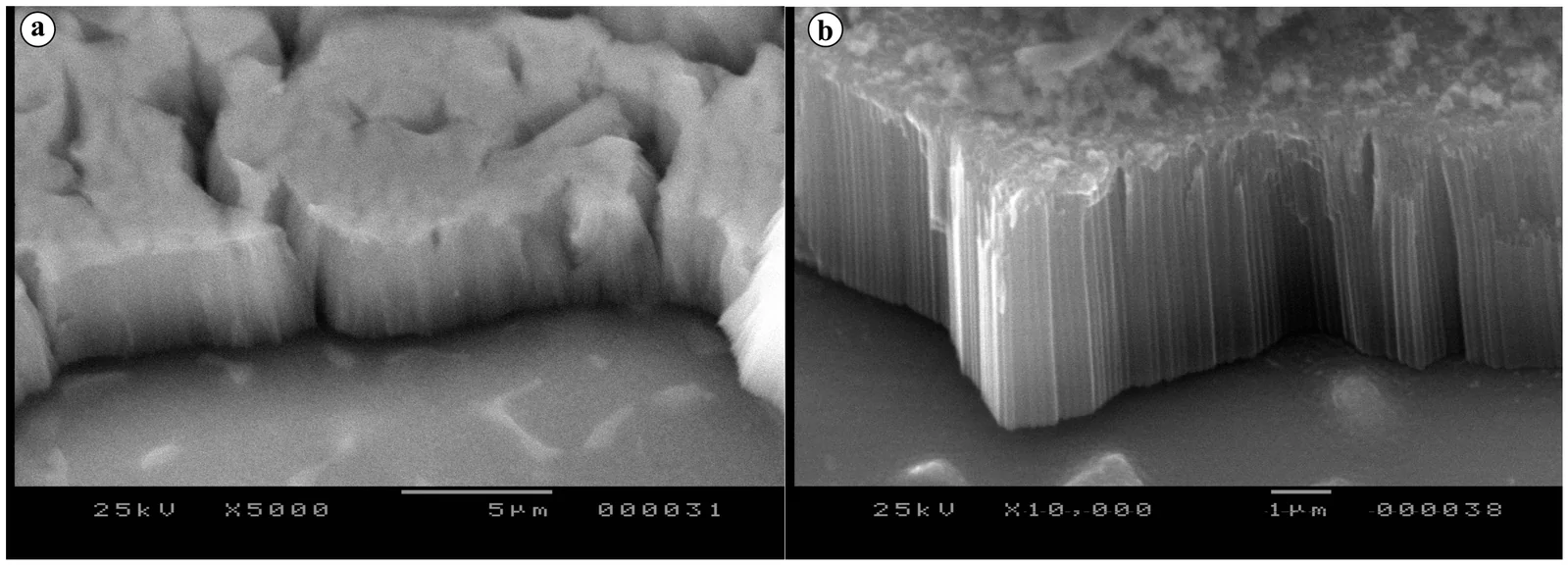
Representative cross-sectional SEM micrographs of TiO_2_ nanotube arrays synthesized at 50 V for 30 min (recorded at a 45° stage tilt angle): (**a**) CP-Ti and (**b**) Ti6Al4V.

Representative cross-sectional SEM micrographs obtained at an intermediate anodization potential (50 V) for both substrates are displayed in Figure 5.

As shown in Figure 5, both substrate materials promote the growth of densely packed, highly aligned nanotube arrays oriented perpendicular to the underlying metallic substrate. The low-magnification cross-sectional view of the CP-Ti sample (Figure 5a) visibly illustrates the continuity of the anodic layer along the mechanically fractured edge, confirming strong mechanical adhesion to the titanium substrate. At higher magnification (Figure 5b, Ti6Al4V), the exposed profile reveals smooth, parallel individual tube channels extending linearly across the entire film thickness, exhibiting well-defined tubular walls without structural collapse or major lateral inter-tubular voids. The absence of internal void collapse and the presence of smooth, parallel channels are essential for pharmaceutical applications. Such unobstructed, high-aspect-ratio capillary structures ensure that liquid therapeutic agents can fully infiltrate the reservoir down to the barrier layer, maximizing the total volume of encapsulated drugs [12]. 

To provide a comprehensive quantitative synthesis of the electrochemical anodization process, the geometric parameters, statistical metrics derived from the Gaussian fitting, mass loss data, and cross-sectional layer thicknesses are summarized in Table 1.

As reported in Table 1, increasing the anodization potential from 40 V to 60 V significantly affects the pore growth and has a profound, proportional effect on both the lateral and vertical dimensions of the synthesized oxide arrays:

- **Nanostructure Diameter Kinetics:** The mean inner pore diameter systematically increases as voltage increases (*p* < 0.05). For CP-Ti, the average diameter expands from 59 ± 10 nm (40 V) to 77 ± 15 nm (50 V) and reaches 88 ± 19 nm at 60 V. Similarly, for the Ti6Al4V alloy, the mean values increase from 56 ± 13 nm (40 V) to 70 ± 13 nm (50 V) and 96 ± 15 nm (60 V). The strong close alignment among the average, median, and mode values across nearly all conditions confirms the symmetrical nature of the pore distributions modeled by the Gaussian curves.
- **Layer Thickness and Growth Rate:** The cross-sectional layer thickness demonstrates an even more dynamic, non-linear growth trajectory driven by the higher electric field strength. At 40 V, oxide layers with thicknesses of 3.43 µm (CP-Ti) and 3.83 µm (Ti6Al4V) are obtained. Increasing the potential to 50 V yields layer lengths of 4.57 µm and 5.11 µm, respectively. At 60 V, the field-assisted oxidation kinetics accelerate significantly, resulting in thick tubular films of 9.95 µm for CP-Ti and 10.57 µm for Ti6Al4V. Across all testing conditions, the Ti6Al4V alloy consistently exhibits slightly higher vertical growth rates than pure titanium. This exponential increase in tube length at 60 V fundamentally transforms the functional capability of the surface. By expanding the vertical depth to approximately 10 µm, the three-dimensional storage capacity of the matrix is vastly augmented, effectively converting the implant coating into a high-volume, sustained-release drug depot.
- **Mass Loss Dynamics (∆m):** The recorded mass loss values reflect the balance between field-assisted dissolution and tube growth. The highest mass loss occurs at intermediate potential (0.7 mg for CP-Ti at 50 V), while at 60 V the values drop significantly to 0.2 mg (CP-Ti) and 0.1 mg (Ti6Al4V), indicating that rapid vertical oxide growth dominates over chemical etch-dissolution into the electrolyte at higher potentials.

Importantly, comparative measurements after thermal annealing at 450 °C confirmed that the geometric metrics (pore diameters and layer thicknesses) presented in Table 1 remained fully preserved, verifying the structural robustness of the anatase nanotube arrays.

Furthermore, the achieved geometric dimensions—particularly the 50–100 nm pore diameters and up to ~10 µm layer thicknesses at higher potentials—are in excellent agreement with classic growth models reported for fluoride-based organic electrolytes, confirming the highly reproducible nature of our optimized matrices compared to established literature data [13].

### 3.2. Surface Roughness Analysis

To complement the microstructural evaluation and understand the influence of surface micro-topography on potential biomedical applications, quantitative surface roughness measurements (R_a_, arithmetical mean deviation of the assessed profile) were carried out using a Mitutoyo SJ-310 profilometer (Mitutoyo Corporation, Kawasaki, Japan). Measurements were performed across four distinct surface states for both CP-Ti and Ti6Al4V substrates: as-polished baseline, as-anodized (40 V, 50 V, and 60 V), polished post-annealing (450 °C), and anodized post-annealing (450 °C). Each experimental condition was evaluated in triplicate (n = 3) to guarantee statistical reliability. The recorded roughness parameters are quantitatively summarized in Table 2.

As detailed in Table 2, the initial mechanical polishing process yielded exceptionally smooth baseline surfaces for both CP-Ti (R_a_ = 0.046–0.048 µm) and Ti6Al4V (R_a_ = 0.046–0.047 µm), providing a highly uniform substrate matrix prior to anodization.

Following electrochemical anodization, a distinct potential-dependent increase in surface roughness is observed across both substrate materials:

- **Effect of Anodization Potential:** As the applied potential increases from 40 V to 50 V and 60 V, the R_a_ values systematically increase (*p* < 0.05). For CP-Ti, the surface roughness increases from 0.066 µm (40 V) to 0.084 µm (50 V) and reaches a peak of 0.138 µm at 60 V. A matching trend is recorded for the Ti6Al4V alloy, where R_a_ increases from 0.070 µm (40 V) to 0.083 µm (50 V) and 0.120 µm (60 V). This gradual improvement directly correlates with the lateral pore widening observed in Figure 3 and Table 1, as larger pore openings and thicker tube walls introduce greater vertical topography variation. In the context of localized drug delivery, this systematic increase in surface roughness is highly advantageous. A rougher topographical profile, driven by wider pore openings, significantly increases the effective surface area available for the physical adsorption of therapeutic molecules, thereby providing an expanded structural interface designed to maximize the overall drug loading capacity of the matrix [14].
- **Effect of Thermal Annealing (450 °C):** Thermal treatment induces a secondary, statistically significant increase in surface roughness across all samples (*p* < 0.05). For the un-anodized polished substrates, annealing at 450 °C leads to a consistent baseline elevation up to R_a_ ≈ 0.108–0.111 µm due to native thermal oxidation and crystal domain growth. For the anodized nanotube arrays, thermal annealing further enhances the final roughness, reaching maximum values of 0.208 µm (CP-Ti at 60 V) and 0.158 µm (Ti6Al4V at 60 V). This structural change is attributed to the amorphous-to-crystalline (anatase) phase transformation, accompanied by localized atomic rearrangements along the tubular oxide walls. Beyond merely altering topography, this crystallization-induced roughness plays a pivotal role in the thermodynamic stabilization of the surface. For pharmaceutical applications, this stable, crystalline rough interface provides superior anchoring points for fluid infiltration, which is essential to withstand the aggressive capillary forces generated during the drying phase of therapeutic liquid formulations.

Overall, the combination of high-voltage anodization (60 V) and thermal annealing effectively transforms the mirror-polished metallic surfaces into structured nanomorphologies with enhanced micro-roughness, which is widely recognized as highly beneficial for promoting cell attachment and protein absorption in biomaterial interfaces. Consequently, this optimized micro-roughness not only facilitates biological integration but also creates a highly receptive physical template for retaining pharmaceutical coatings and promoting sustained, localized drug release.

### 3.3. Wettability and Surface Energy Assessment

**Figure 6:**
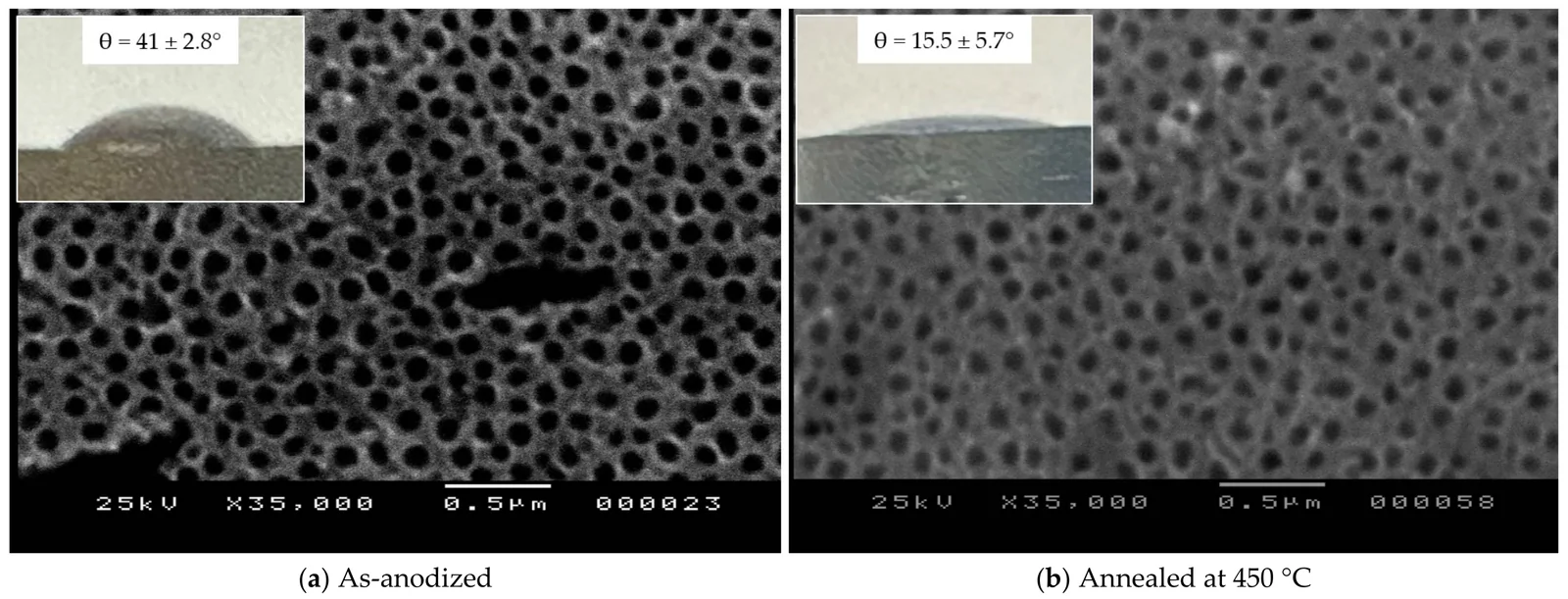
Surface nanomorphology and inset water contact angles (θ) of TiO_2_ nanotubes formed on CP-Ti at 60 V.

**Figure 7:**
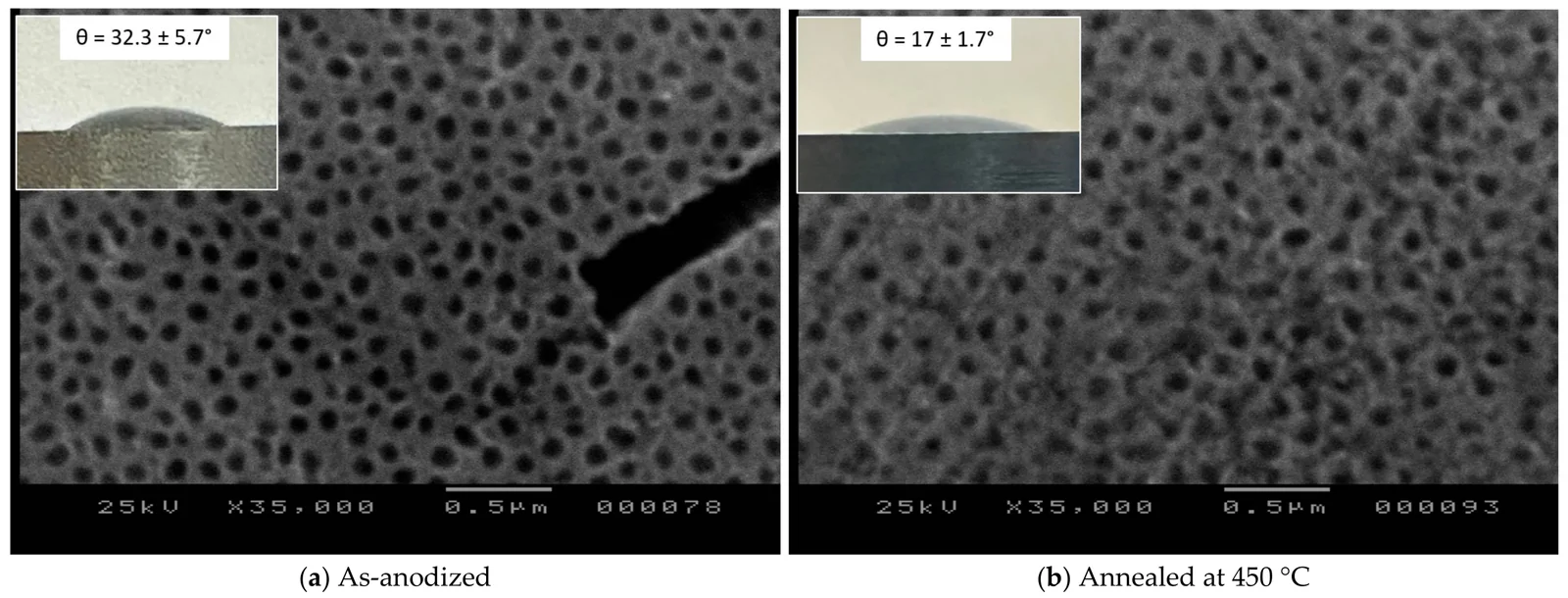
Surface nanomorphology and inset water contact angles (θ) of TiO_2_ nanotubes formed on Ti6Al4V at 60 V.

Surface wettability plays a decisive role in biological interfaces, directly influencing protein adsorption, cell adhesion, and subsequent tissue integration. To evaluate the hydrophilic character of the synthesized nanostructures, static distilled water contact angle (θ) measurements were realized. The surface nanomorphology correlated directly with the contact angle profiles for CP-Ti and Ti6Al4V substrates at 60 V is presented in Figure 6 and Figure 7, respectively. The complete quantitative evolution of surface wettability across all anodization potentials (40 V, 50 V, and 60 V)—both in the as-anodized state and after thermal annealing at 450 °C—is summarized in Table 3.

As demonstrated in Figure 6 and Figure 7, and Table 3, both the applied anodization potential and the subsequent thermal treatment have a significant impact on the surface contact angle:

- **Influence of Anodization Voltage (As-Anodized State):** In the unannealed condition, increasing the anodization potential systematically enhances surface hydrophilicity (*p* < 0.05). For CP-Ti, the contact angle decreases from 78.5 ± 0.7° (40 V) to 61.5 ± 7.8° (50 V) and drops to 41.0 ± 2.8° at 60 V (Figure 6a). A matching trend is recorded for the Ti6Al4V alloy, where θ decreases from 68.5 ± 3.9° (40 V) to 58.5 ± 0.3° (50 V) and reaches 32.3 ± 5.7° at 60 V (Figure 7a). This potential-driven transition toward higher hydrophilicity directly correlates with the morphological broadening of the inner pore diameters (Table 1) and the elevated micro-roughness (R_a_, Table 2). Larger pore openings facilitate liquid penetration into the nanotubular cavities via capillary action, consistent with the Wenzel wetting model [5]. In the context of pharmaceutical loading, this enhanced capillary action is highly advantageous. The ability of the wider, rougher 60 V pores to actively draw in liquids ensures that aqueous therapeutic solutions can deeply penetrate the reservoir without trapping air bubbles, thereby providing the essential physical mechanism to maximize the utilized encapsulation volume [15].
- **Influence of Thermal Annealing (450 °C):** Thermal crystallization drastically alters the surface chemical behavior, triggering a statistically significant shift toward superhydrophilic states (θ < 20°) across all conditions (*p* < 0.05). Following annealing at 450 °C, contact angles drop sharply to 15.5 ± 5.7° for CP-Ti at 60 V (Figure 6b) and to 17.0 ± 1.7° for Ti6Al4V at 60 V (Figure 7b). Across all potentials, the annealed contact angles remain between 15.5° and 21.0°. This pronounced enhancement in wettability is primarily attributed to the phase transformation from the amorphous anodic film into the crystalline anatase structure, accompanied by the accumulation of surface hydroxyl groups (–OH) and the thermal degradation of residual organic carbon species from the electrolyte [16]. For a targeted drug delivery system, this superhydrophilic state is a key functional requirement. It guarantees the immediate and homogeneous wetting of the implant surface by biological fluids upon implantation, ensuring the fundamental physical conditions necessary for a reliable, predictable activation of the therapeutic release profile.

The combination of high-potential anodization (60 V) and 450 °C thermal annealing successfully converts the baseline metallic substrates into highly hydrophilic surfaces (θ ≈ 15–17°), which is highly desirable for accelerating osseointegration in biomedical implant applications. Beyond osseointegration, this engineered superhydrophilicity provides the optimal interfacial energy required for the rapid capillary infiltration of liquid therapeutic agents, establishing these nanostructured matrices as robust, structurally optimized frameworks for high-capacity drug delivery applications.

This drastic shift to a superhydrophilic state post-annealing strongly aligns with previous observations in the literature, which consistently demonstrate that the transition to a crystalline anatase phase yields highly wettable TiO_2_ surfaces, considered optimal for both orthopedic integration and rapid fluid infiltration [17].

### 3.4. Fluid Exposure and Capillary Stress Resistance

To evaluate the ability of the developed TiO_2_ nanotube arrays as local drug-delivery carriers, fluid exposure and capillary stress resistance tests were performed. Magnesium sulfate (MgSO_4_) aqueous solution was selected as a model inorganic salt compound to simulate the infiltration, surface loading, and drying dynamics of a liquid drug formulation within the tubular nanopores. Validating the mechanical stability under these simulated conditions is a fundamental prerequisite for pharmaceutical translation. Real-world therapeutic loading inevitably involves solvent evaporation, generating intense localized capillary forces that the nanostructured reservoir must withstand without catastrophic failure.

The drug-loading process was initially attempted on as-anodized (amorphous) CP-Ti and Ti6Al4V samples. To facilitate rapid liquid infiltration and solvent evaporation, two distinct thermal drying strategies were tested: (i) pre-heating the substrates at 60 °C before droplet deposition followed by oven drying, and (ii) heating the substrates from the bottom via a heated iron plate to accelerate upward evaporation. In both unannealed cases, although the initial wetting and infiltration appeared successful, severe structural failure occurred within 24 h. The high capillary stresses induced by solvent evaporation within the high-aspect-ratio amorphous nanotubes, combined with the inherently low interfacial adhesion of the unannealed anodic layer, resulted in complete delamination and peeling of the nanotube film from the metallic substrates [9]. This catastrophic delamination clearly disqualifies the as-anodized, amorphous matrices from functioning as reliable drug carriers. If such a failure were to occur in vivo, it could lead to uncontrolled burst release and potential toxicity, highlighting the imperative need for microstructural stabilization prior to pharmaceutical loading.

To overcome this structural degradation, the MgSO_4_ solution loading was subsequently performed on samples that had undergone thermal annealing at 450 °C. The post-crystallization state exhibited a remarkable enhancement in mechanical robustness and adhesion strength. Following the same fluid exposure and drying sequence, the 450 °C annealed nanotube layers remain firmly attached to the substrate without any film delamination.

**Figure 8:**
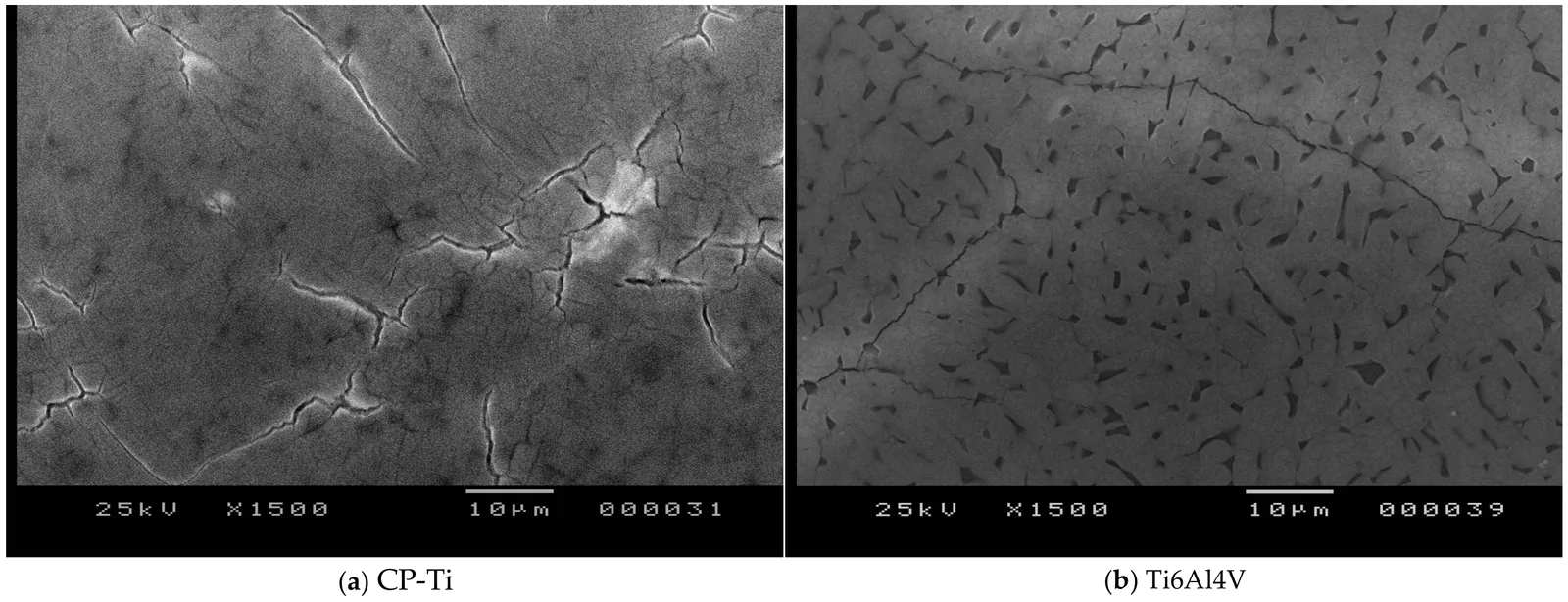
SEM micrograph (1500×) of 450 °C annealed TiO_2_ nanotubes after MgSO_4_ loading, showing surface micro-cracking without film delamination.

Scanning electron microscopy (SEM) inspection at 1500× magnification (Figure 8) revealed the formation of localized micro-cracks across the surface of the drug-loaded annealed layers for both CP-Ti (Figure 8a) and Ti6Al4V (Figure 8b).

However, as evidenced by Figure 8, these micro-cracks were purely superficial and did not compromise the overall structural integrity or cause film detachment. The transition from total film failure in the amorphous state to localized micro-cracking in the crystalline state underscores the essential role of thermal annealing at 450 °C (anatase crystallization) in providing sufficient capillary stress resistance. Nevertheless, from a clinical orthopedic perspective, these micro-cracks present a severe biomedical risk. We fully acknowledge that structural cracking in an implant coating can lead to particulate shedding in vivo, which is a well-documented primary driver of macrophage activation, foreign body immune responses, and ultimately, aseptic loosening of the implant [18]. Therefore, these defects cannot be dismissed as beneficial micro-channels. Future optimization must strictly focus on mitigating this micro-cracking—either by refining post-loading drying kinetics or by employing biocompatible polymer capping layers to seal the matrix—ensuring safe clinical translation without the risk of particulate debris [19].

### 3.5. Parameter-Property Performance Matrix

To synthesize the comprehensive experimental findings across all 12 sample configurations and establish practical engineering guidelines for biomedical applications, a holistic parameter-property performance matrix was formulated (Table 4). This matrix systematically correlates the primary processing variables substrate chemistry (CP-Ti vs. Ti6Al4V), anodization potential (40 V, 50 V, and 60 V), and thermal treatment (450 °C)—with key physical metrics (D, θ, and capillary drying stability) to define targeted biomedical functionality. By bridging the gap between electrochemical synthesis parameters and structural robustness, this matrix serves as a direct design tool for formulating the structural foundation of advanced, implantable drug delivery platforms intended for tailored to specific kinetic release profiles.

As outlined in Table 4, the performance matrix reveals distinct functional regimes based on structural dimensions and thermal processing:

- **As-Anodized vs. Annealed Integrity:** Regardless of substrate material or anodization voltage, all unannealed (as-anodized) films exhibit severe capillary stress failure (delamination) upon liquid exposure. Consequently, these amorphous states are restricted to fundamental dry surface characterizations and baseline model substrates. In a pharmaceutical context, this highlights that thermal crystallization is not merely an optional post-treatment, but an absolute necessity for creating viable, fluid-resistant architectural templates capable of surviving the rigorous drying phases of clinical formulations.
- **Low-to-Medium Potential Regimes (40 V and 50 V with 450 °C Annealing):** Thermal annealing secures structural adhesion while preserving smaller nanotube diameters (D ≈ 56–77 nm). The combination of hydrophilic surface chemistry (θ ≈ 16.5–21.0°) and restricted pore volumes makes the 40 V and 50 V annealed arrays structurally ideal for developing sustained/slow drug release coatings and general orthopedic implants. These narrower matrices are designed to act as restrictive capillary networks, which theoretically impede the rapid outward diffusion of encapsulated molecules. Therefore, they represent the optimal geometric design choice when prolonged, strictly controlled therapeutic dosing is the clinical objective required to prevent local inflammation or infection.
- **High-Potential Optimal Regime (60 V with 450 °C Annealing):** The 60 V anodization paired with 450 °C crystallization delivers maximum inner pore diameters (D ≈ 88–96 nm), superhydrophilicity (θ ≈ 15.5–17.0°), and stable film adhesion. This configuration offers the highest structural potential as a high-capacity drug delivery reservoir and for accelerating osseointegration on both CP-Ti and Ti6Al4V implants. Ultimately, this optimal regime perfectly aligns with the fundamental physical requirements of advanced pharmaceutics. By maximizing the geometric void volume while ensuring superhydrophilic fluid infiltration and mechanical robustness, the 60 V annealed arrays provide a state-of-the-art physical framework designed for localized, high-dose therapeutic interventions directly at the bone-implant interface.

### 3.6. Future Research Directions

While the present study successfully establishes a foundational relationship between electrochemical anodization parameters, thermal annealing, and structural stability for TiO_2_ nanotube arrays on CP-Ti and Ti6Al4V substrates, some features remain open for future investigation and need to be wholly clarified. 

First, further optimization of the thermal processing protocols—specifically fine-tuning heating rates, dwell times, and cooling atmospheres—will be systematically explored to eliminate the residual micro-cracking observed during high-capacity drug loading while maintaining the desired anatase crystallinity and superhydrophilicity.

Second, to achieve higher precision and more robust microstructural insights, future analytical workflows will incorporate advanced characterization techniques such as scanning transmission electron microscopy (STEM) and transmission electron microscopy (TEM). These methods will enable atomic-resolution evaluation of the nanotube barrier layers, interfacial oxides, and phase transformations.

Third, to transition these structured surfaces from model systems into clinically viable therapeutic platforms, current efforts are directed toward refining the active drug-loading methodologies, incorporating biocompatible polymer capping layers, and evaluating empirical in vitro release kinetics using relevant pharmaceutical agents.

Finally, to address the long-term biocompatibility and mechanical limitations associated with conventional titanium alloys (such as elastic modulus mismatch and potential ion cytotoxicity), future research will expand this anodization and drug-delivery roadmap toward advanced, low-modulus, beta-type titanium alloys developed and described in [20]. Specifically, novel multi-element systems such as Ti-10Nb-10Zr-5Ta, Ti-20Nb-20Zr-4Ta, and Ti-29.3Nb-13.6Zr-1.9Fe will be further investigated to determine how their unique phase compositions and electrochemical behaviors influence nanotube growth kinetics, fluid infiltration, and localized therapeutic performance [20].

## 4. Conclusions

In this study, highly ordered TiO_2_ nanotube arrays were successfully engineered on CP-Ti and Ti6Al4V alloy substrates by tuning electrochemical anodization parameters and applying thermal annealing. The findings demonstrate that anodization potential effectively dictates structural dimensions, surface roughness, and wettability, with CP-Ti exhibiting slightly more uniform pore growth compared to the dual-phase Ti6Al4V substrate. Thermal crystallization into the anatase phase proved to be an absolute prerequisite for pharmaceutical applications. While amorphous layers suffered from catastrophic delamination under capillary drying stresses, 450 °C annealing provided robust interfacial adhesion and induced a stable superhydrophilic surface state. This structural stabilization not only ensures mechanical resilience against fluid exposure, but also facilitates the rapid, homogeneous capillary infiltration of liquid formulations into the nanoporous network.

Ultimately, the combination of high-potential anodization (60 V) and thermal annealing emerges as the optimal engineering configuration, offering a maximized void volume, high wettability, and strong mechanical integrity. These optimized nanostructured matrices provide a robust architectural foundation for localized, high-capacity drug delivery and accelerated osseointegration on orthopedic and dental implants. While this study establishes a highly controlled structural and morphological roadmap for customizing TiO_2_ nanotube arrays, future quantitative in vitro investigations are required to evaluate the empirical drug-loading capacities and specific kinetic release profiles of these engineered matrices.

## Figures and Tables

**Table 1:** Process parameters, mass loss, and statistical nanostructure diameter distribution parameters of TiO_2_ nanotube arrays.

Process Parameters			Material	Mass Loss ∆m (mg)	Nanostructure Diameter D (nm)					Layer Thickness (µm)
Electrolyte	Potential U (V)	Time T (min)			Average	Standard Deviation	Range	Median	Mode	
0.5 wt% NH_4_F + 2 wt% H_2_O + EG	40	30	CP-Ti	0.5	59	10	41–83	58	49	3.43
			Ti6Al4V	0.4	56	13	32–85	55	46	3.83
	50		CP-Ti	0.7	77	15	42–99	80	62	4.57
			Ti6Al4V	0.5	70	13	44–103	69	73	5.11
	60		CP-Ti	0.2	88	19	49–120	90	90	9.95
			Ti6Al4V	0.1	96	15	62–122	100	89	10.57

Statistically significant difference in nanostructure diameter across the applied potentials (40 V, 50 V, 60 V) for both materials (ANOVA, *p* < 0.05).

**Table 2:** Surface roughness (R_a_) evolution of CP-Ti and Ti6Al4V substrates before and after electrochemical anodization and subsequent thermal annealing at 450 °C.

Material	Potential (V)	Surface Roughness, Ra (µm)			
		As-Prepared		Thermally Annealed (450 °C)	
		Polished	Anodized	Polished	Anodized
CP-Ti	40	0.046	0.066	0.111	0.124
Ti6Al4V	40	0.046	0.070	0.108	0.130
CP-Ti	50	0.047	0.084	0.110	0.130
Ti6Al4V	50	0.046	0.083	0.109	0.130
CP-Ti	60	0.048	0.138	0.111	0.208
Ti6Al4V	60	0.047	0.120	0.109	0.158

Statistically significant differences in surface roughness (R_a_) were observed as a function of both the applied anodization potential and the thermal annealing process (ANOVA, *p* < 0.05).

**Table 3:** Contact angle (θ) of TiO_2_ nanotube arrays formed on substrates before and after thermal annealing at 450 °C.

Material	Potential (V)	As-Anodized	Annealed at 450 °C
CP-Ti	40	78.5 ± 0.7°	17.5 ± 8.3°
Ti6Al4V	40	68.5 ± 3.9°	21 ± 2.6°
CP-Ti	50	61.5 ± 7.8°	16.5 ± 3.9°
Ti6Al4V	50	58.5 ± 0.3°	19 ± 2.8°
CP-Ti	60	41 ± 2.8°	15.5 ± 5.7°
Ti6Al4V	60	32.3 ± 5.7°	17 ± 1.7°

Statistically significant decrease in contact angle following thermal annealing across all groups, as well as a significant voltage-dependent decrease in the as-anodized state (ANOVA, *p* < 0.05).

**Table 4:** Parameter-Property Performance & Application Matrix.

Substrate	Voltage (V)	State	D (nm)	θ (°)	Capillary Stability	Recommended Application
CP-Ti	40	As-anodized	59	78.5 ± 0.7	Delaminated	Fundamental dry surface studies
		450 °C		17.5 ± 8.3	Stable	Sustained/Slow drug release coatings
	50	As-anodized	77	61.5 ± 7.8	Delaminated	Fundamental dry surface studies
		450 °C		16.5 ± 3.9	Stable	Moderate-release orthopedic implants
	60	As-anodized	88	41 ± 2.8	Delaminated	High-roughness dry model substrates
		450 °C		15.5 ± 5.7	Stable	High-capacity drug reservoir & Rapid osseointegration
Ti6Al4V	40	As-anodized	56	68.5 ± 3.9	Delaminated	Fundamental dry surface studies
		450 °C		21 ± 2.6	Stable	Sustained/Slow drug release coatings
	50	As-anodized	70	58.5 ± 0.3	Delaminated	Fundamental dry surface studies
		450 °C		19 ± 2.8	Stable	Moderate-release load-bearing implants
	60	As-anodized	96	32.3 ± 5.7	Delaminated	High-roughness dry model substrates
		450 °C		17 ± 1.7	Stable	High-capacity drug reservoir & Rapid osseointegration

## Data Availability

The raw and processed data required to reproduce these findings cannot be shared at this time, as they also form part of an ongoing study.
